# Reintegration of Brewers Spent Grains in the Food Chain: Nutritional, Functional and Sensorial Aspects

**DOI:** 10.3390/plants10112504

**Published:** 2021-11-18

**Authors:** Anca Corina Fărcaș, Sonia Ancuța Socaci, Maria Simona Chiș, Oana Lelia Pop, Melinda Fogarasi, Adriana Păucean, Marta Igual, Delia Michiu

**Affiliations:** 1 Department of Food Science, Faculty of Food Science and Technology, University of Agricultural Sciences and Veterinary Medicine of Cluj-Napoca, 3-5 Mănăştur Street, 400372 Cluj-Napoca, Romania; sonia.socaci@usamvcluj.ro (S.A.S.); oana.pop@usamvcluj.ro (O.L.P.); 2 Department of Food Engineering, Faculty of Food Science and Technology, University of Agricultural Sciences and Veterinary Medicine of Cluj-Napoca, 3-5 Mănăştur Street, 400372 Cluj-Napoca, Romania; melinda.fogarasi@usamvcluj.ro (M.F.); adriana.paucean@usamvcluj.ro (A.P.); delia.michiu@usamvcluj.ro (D.M.); 3 Food Investigation and Innovation Group, Food Technology Department, Universitat Politècnica de València, Camino de Vera s/n, 46022 Valencia, Spain; marigra@upvnet.upv.es

**Keywords:** brewers spent grain, volatile compounds, minerals, sensorial analysis

## Abstract

Nowadays, the pandemic situation has encouraged the idea of sustainable healthy foods leading to new trends in food consumption. Brewers spent grain (BSG) represents a potential functional food rich in fiber, protein, lipids, mineral and phenols that needs to be further exploited. In this vein, five different BSG types were collected from local breweries and valorized in cookies manufacturing. Thus, proximate composition (protein, minerals, lipids, ash, crude fiber and carbohydrates) was analyzed using AACC (American Association of Cereal Chemists) methods, DPPH (2,2-Diphenyl-1-picrylhydrazyl), and Folin Ciocalteu methods were used to determined antioxidant activity and total phenols, while minerals and aroma volatile compounds were performed using inductively coupled plasma optical emission spectrometry (ICP-OES) and ITEX/GC-MS (in tube extraction gas chromatography-mass spectrometry) respectively. Color and physical characteristics, together with sensorial analysis, were also evaluated. The results highlighted a significant difference between BSG samples, mainly from the total phenols, antioxidant activity and aroma volatile compounds point of view. BSG volatiles compounds from the aldehydes group such as 2-methyl-propanal, 3-methyl-butanal and 2-methyl-butanal were identified also in the final baked goods, leading to a pleasant and appreciated consumers’ taste and aroma. Furthermore, cookies sensorial analysis emphasized that the sample manufactured with BSG from light and dark malt mixture was more appreciated by consumers, attaining the highest hedonic scores.

## 1. Introduction

The world population is expected to reach 9.8 billion people in 2050 while millions of people are starving today. World cereal equivalent food demand is anticipated to be 10.094 million tons in 2030, which will increase to 14.886 million tons in 2050 [1]. Thus, the adoption of policies and actions for sustainable consumption and the transition to a cleaner and circular bioeconomy is highly required [2]. Furthermore, the pandemic situation has changed consumers’ dietary habits, and their attention was directed towards healthier and balanced diets [3]. Moreover, the pandemic situation has significantly increased food insecurity, and the nutritional indicator has been affected mainly by consumer behavior changes [4]. In this way, the researcher’s attention is focused on the reduction of agro-industrial waste with maximizing the conversion of the by-products into higher added-value products [2]. Nowadays, the exploitation of agro-industrial waste from economic, technological and environmental standpoints represents new researchers’ tools [5].

Around 1.91 billion hectoliters of beer were produced worldwide in 2019, with beer being on fifth place in the world’s beverage consumption [6]. The industrial brewing process is one of the biggest agro-industrial waste producers, with a high impact on the environment and therefore on society. Indeed, for the production of 1000 tons of beer, a total amount ranging from 137 to 173 tons of solid waste is generated [6]. The solid waste is mainly composed of brewers spent grain (BSG), spent hops and trub, spent yeast and barley malt rootlets [7].

In a recent publication of Naibaho et al. [8], it was highlighted that the EU brewing industry produces about 3.4 million tons of BSG annually, mainly being used as animal food, organic fertilizer and for bricks production. From a biotechnological perspective, other applications of the BSG biomass could be as a substrate for ethanol production due to its high cellulose concentration, as well as fuels, enzyme production, proteins concentrate and the manufacturing of many other value-added products [8,9]. The importance of using BSG should also be highlighted from an economic point of view, as demonstrated by Olawoye et al. [10] who stated that the cost of BSG is 0.0335 US$/kg or that it has no cost [11], being available all through the year.

BSG is mainly defined as the brewing process by-product, a result from the mashing and filtration stages, being mainly composed of seed coat–pericarp–husk layers of malted barley [7,12]. Certain malting processes use a wide variety of source ingredients to produce different malt varieties. On these considerations, various BSG components were categorized into two major groups: lighter malts (pilsen, melano, melano 80 and carared) and darker malts (chocolate and black), according to Moreira et al. [12]. Pilsner malt is often used as a foundation malt in any and all beer types, producing light-colored, clean and malt extracts. Caramel malt is generated from fresh malt, which is created by heating wet germinated barley at regulated heat, allowing the starch to change into caramelized sugars. Melano malt is a distinctive type that is gently dried; the rising temperature enables melanoidins to develop throughout the kilning procedure. Chocolate malt has the same kind of qualities as black malt, but as it is roasted a lesser length of time and at lower-end degrees, a few of the harsh tastes of black malt are not quite as apparent [13].

BSG represent 85% of the solid waste amount brewing process, and is a rich source in fibers (40–70%), proteins (19–30%), minerals (2–5%), lipids (about 10%), vitamins and phenols (0.7–2%) [2,6]. BSG contains an important amount of amino acids such as threonine (3.5 g/100 g), isoleucine and valine, phenylalanine (5.5 g/100 g), lysine and leucine [14]. Fibers are represented by lignin, cellulose, hemicellulose, arabinoxylans and β-glucan, which are involved in the serum cholesterol regulation and the prevention of gastrointestinal disorders and diabetes, and could also be successfully used in the treatment of ulcerative colitis [2]. Furthermore, from the phenols group, hydroxycinnamic acids are claimed to have a high in vitro antioxidant effect, similar with the well-studied ascorbic acid and α-tocopherol [11]. Nevertheless, its chemical composition could be highly varied owing to multiple factors, such as the type of barley used, the harvesting time, malting and mashing conditions and the secondary raw materials used in the brewing process [6].

Cookies are defined as popular confectionary product manufactured with wheat flour, that have high caloric value and are low in protein and fiber content [15], commonly called biscuits in some regions [16]. Cookie consumption increased worldwide, mainly due to their extensible shelf life, convenience and urbanization [10], but their nutritional value should be improved. In this view, BSG could represent an affordable and valuable raw material, able to increase the cookies’ nutritional value.

The aim of the present research is to evaluate the variability of five different brewers spent grains by-products and their impact on the nutritional composition, functional properties and sensorial profile of developed food products.

## 2. Results & Discussion

### 2.1. BSG Samples Proximate Composition

Proximate composition of the raw materials is displayed in Table 1. Protein content of the wheat flour (WF) was the lowest (10.03%), while BSG samples’ protein content ranged between 24.00 to 25.80%. According to a large body of literature, BSG protein content could vary between 24.0 to 15.2%, [17] while Shih et al. [18] mentioned a protein value content ranging from 14.2 to 31 g/100 g. Moisture parameter did not differ significantly between BSG samples (7.21% to 7.52%), while WF value (14.5%) was significantly different (*p* < 0.05).

Regarding the BSG total lipids content (Table 1), a significant difference (*p* < 0.05) was highlighted between BSG samples, with the smallest amount being exhibited by BSG3 (5.96%), while BSG2 recorded a value of 7.05% total lipids. The results are in line with those mentioned in the literature, with Fărcaș et al. [7] reporting a BSG lipid value ranging between 5.40 to 11%. Triacylglycerols, free fatty acids, diacylglycerols, monoacylglycerols, phospholipids and steroid compounds were reported by Fărcaș et al. [7] as being predominant BSG lipid classes.

The total ash content in the present study ranged between 2.85 and 3.46%. This result aligned with previous studies, where ash was reported in a range of 2.4–4.6% by Mussatto et al., [17] between 2.68–4.10 g/100 g by Shih et al. [18], and in a total amount between 2.2–7.9%, as mentioned by Aliyu et al. [19].

With respect to crude fiber content (Table 1), the differences between BGS samples were not significantly different (*p* < 0.05), while WF registered the smallest fiber amount. The main BSG fibers are cellulose, hemicellulose and lignin, and registered values between 60–70 g/100 g d.w., as emphasized by Shih et al. [18]. Amoriello et al. [6] reported arabinoxylans and beta-glucan as the main representative of soluble and insoluble fibers involved in the prevention of different gastro-intestinal disorders, the regulation of serum cholesterol and in the treatment of ulcerative colitis. Furthermore, beta-glucan was reported as a potential prebiotic with a positive influence on gastrointestinal tract, while arabinoxylans could contribute to glucose reduction [9]. Moreover, there is a daily recommendation of 25 g of dietary fiber, as emphasized by Ajanaku et al. [16]. The variations in BSG samples chemical composition could be related to several factors, such as barley variety, type of used malt, harvesting conditions, the brewing process and the time when BSG is taken out from the brewing process [20].

Regarding the raw materials total phenols content (Table 1), the highest values were reached by BSG3, followed by BSG4 and BSG1, with values of 258.12 mg GAE/100 g f.w, 242.56 mg GAE/100 g f.w. and 236.31 mg GAE/100 g fw, respectively. The high amounts of polyphenols could be justified by the fact that the majority of the polyphenolic content are identified in the husk barley grain, which represent the main component of BSG [17]. In the same vein, Socaci et al. [21] underlined that BSG could be considered as a rich source in phenolic compounds, being mainly composed of coat-pericarp-husk layers barley grain. Moreover, using scanning electron microscopy, Ktenioudaki et al. [22] emphasized once again that BSG structure is formed mainly of barley husks and fiber filaments, and the idea was straightened by Fărcaș et al. [23], who defined BSG as being mainly composed of barley grain husk, with only minor fractions of pericarp and barley endosperm.

A higher total phenols BSG content was reported also by Moreira et al. [11], who emphasized that BSG obtained from a malt kilning temperature of 160 °C contained a higher amount of total phenols, compared with BSG obtained from a malt kilning temperature higher than 200 °C. Contrarily, the high kilning temperature could be able to break up the acetal, ether and esters bonds that entrap phenolics, improving the bioactive compound amount [18]. Another important parameter that could influence the extraction of phenols from BSG is the extraction technique used, considering that BSG could be characterized as a lignocellulosic material that might entrap the phenolic acid in the cell-wall [21].

The difference between BSG total phenols samples could be explained by the type of malt used in the brewing process but also by melanoidins, which are compounds formed during the kilning malt process through Maillard reaction [13]. Melanoidins are defined as brown hydrophilic polymers which are formed in the last stage of Maillard reaction, due to the interaction between proteins and carbohydrates (polyphenols being sometimes also involved) [24,25]. Melanoidins are reported as having antioxidant and antimicrobial properties, prebiotic activity and antihypertensive implications, which are mainly found in dark beer, malt, cocoa, coffee and toast [24]. Furthermore, melanoidins from coffee were claimed to have a positive role in colon cancer pathogenesis [24].

With respect to radical scavenging activity, BSG3 reached the highest extended value (26.77%), followed by BSG4 (24.59%) and BSG1 (24.31%), respectively. BSG extracts from Pilsen and black malt were reported by Moreira et al. [13] as having a strong in vivo antioxidant activity, being able to protect the cells yeast of *Saccharomyces cerevisiae* against oxidative DNA damage.

### 2.2. BSG (Brewer Spent Grains) and WF (Wheat Flour) Color Characteristics

BSG color parameters such as lightness (L* value), redness (a* value) and yellowness (b* value) are displayed in Table 2. CIE color values are calculated using a mathematical model based on a white light source, object and human observer that represent all colors in terms of L* lightness, a* redness-greenness and b* blueness-yellowness [26].

**Figure 1:**
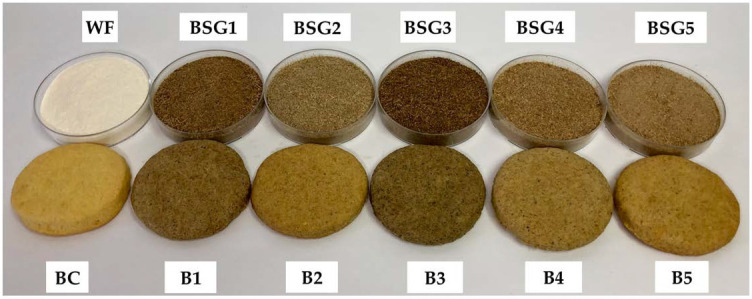
WF (wheat flour) and BSG (brewers spent grains) samples with their corresponding final baked cookies; BC—cookie sample manufactured with 100% WF; B1—cookie sample manufactured with 20% BSG1; B2—cookie sample manufactured with 20% BSG2; B3—cookie sample manufactured with 20% BSG3; B4—cookie sample manufactured with 20% BSG4; B5—cookie sample manufactured with 20% BSG5.

With respect to lightness, significant differences (*p* < 0.05) were identified between WF and BSG samples (Table 2). The lowest value of L* was registered by BSG3 samples, being the most brown sample of all the selected BSGs (Figure 1).

The darker color of BSG3 samples could be explained by the type of malt used and by kilning temperature. At higher temperature reactions such as Maillard, caramelization and pigment degradation could take place and changed the BSG color, reducing its brightness [8]. In the last stage of Maillard reaction, during malt kilning, a brown high molecular compound could be formed, such as melanoidins, which was mainly identified in dark BSG [11,27].

Regarding a* and b* values, the lowest values were recorded by WF, while BSG1 registered the highest a* value and BSG5 the highest b* value (Table 2). Changing in color parameters by addition of BSG have also been reported by a large body of literature [8,15,28,29].

### 2.3. Mineral Determination

Mineral content of the raw materials is presented in Table 3. BSG is a rich source of macrominerals and microminerals, from which calcium (Ca), sodium (Na), magnesium (Mg), phosphorus (P) and zinc (Zn) reached higher values in all BSG samples. This is in line with Waters et al. [30], who showed that BSG is a rich source in minerals such as Ca and Mg, being able to ensure the human daily recommended requirements for Mg (255 mg/day).

With respect to BSG minerals content, Bonifácio-Lopes et al., (Bonifácio-Lopes, Teixeira and Pintado 2020) mentioned that BSG minerals content could vary, as follows: P (1400–600 mg/kg), Ca (2200–3515 mg/kg), Mg (1900–2400 mg/kg), Na (258.1–700 mg/kg). Furthermore, Naibaho et al. [8] justified the difference in BSG mineral content through the type of processing beer, handling and the drying method of the spent grain used, corelated with the treatment during storage. Likewise, a study of BSG from Brazil, Portugal and Ireland emphasized that minerals content could vary, and this is attributed to several factors such as barley type, harvest time, cultivation conditions and type of adjuncts used during wort elaboration [31].

### 2.4. Volatile Aroma Compounds

**Figure 2:**
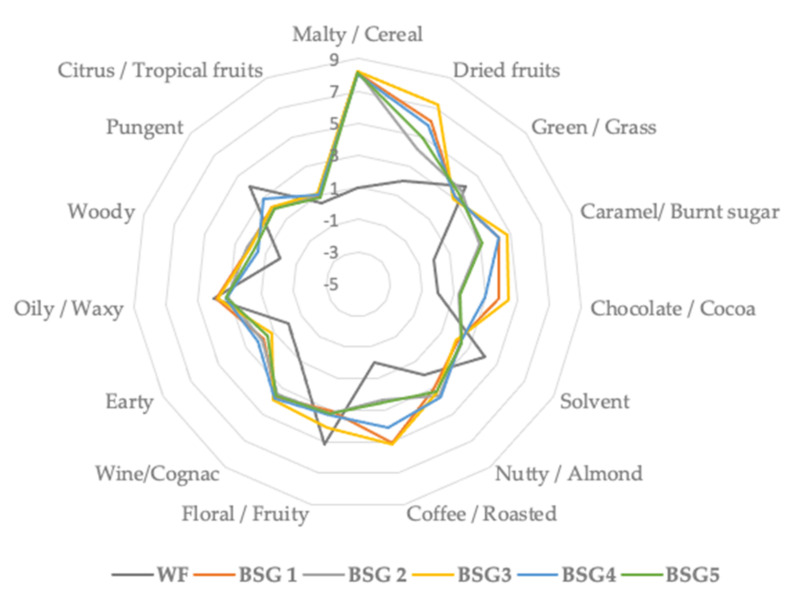
Aroma profile of wheat flour (WF) and different BSG (brewers spent grains) types of main compounds classes identified during GC-MS analysis.

A total number of 26 aroma volatile BSG compounds were separated, identified and displayed in Table 4 and illustrated in Figure 2. For a matter of facilitation, the volatile compounds were divided in alcohols, aldehydes, ketones, terpenes and terpenoids and others. From the alcohols group, the smallest amount was identified in BSG1 (1-pentanol: 2.72%), while BSG5 registered a 1-pentanol amount of 5.36%.

The aldehydes group was mainly represented by 2 methylpropanal, 3-methylbutanal, 2-methylbutanal and hexanal (Table 4), which registered values of 29.8% (BSG 2 sample), 29.05% (in BSG 3), 24.19% in BSG3 and 19.81% (in BSG2 sample), respectively. These results aligned with Ktenioudaki et al. [32], who also identified the aforementioned aldehydes compounds in BSG flour, and explained their provenience through fermentation process and the Maillard reaction. Moreover, the content of aldehydes is a consequence of kilning and roasting malt processes, as a result of lipid enzymolysis and volatilization. The suitable manipulation of the malting process could influence the total amount of aldehydes, as mentioned by Dong et al. [33]. 

Ketones were characterized by 2-heptanone (1.09% for BSG2 sample), ethanone, 1-[4-(1-methylethyl) phenyl]- mostly identified in BSG1 sample and 3-Octen-2-one (0.52% in BSG3 sample), which are responsible for odor perception such as fruity, fungal, buttery, warm spicy, woody, herbaceous and earthy, spicy, herbal, sweet, nutty and fermented, respectively (Table 4). Odor perception such as citrus, fresh, sweet and fresh citrus, terpenic, woody and spicy were mainly exhibited by D-limonene and *p*-cymene, who registered significant amounts in BSG2 and BSG1 samples, respectively.

With respect to WF, a total number of only five aroma volatile compounds were identified, the main representative being 2-methyl-propanal (52.1%), hexanal (37.05%), acetophenone (8.12%) and D-limonene (0.63%); 2-methyl-propanal is responsible for odor perception such as wine, solvent, malty, fruity, hexanal for odors such as intense green, aldehydic, fruity, oily, meantime acetophenone gave odor perception of floral, must, spicy, almond, nutty and D-limonene exhibited odor perception such as citrus, fresh and sweet (Table 4). This result is in line with Fărcaș and al. [34], who showed that WF is a poor source in aroma volatile compounds, based mainly on aldehydes (91.99%) and ketones (8.02%).

### 2.5. Proximate Composition of the Final Baked Goods

Proximate composition of the samples is illustrated in Table 5. BSG addition increased the protein, ash and crude fiber cookies content, being statistically different (*p* < 0.05) from the control sample (Table 5). The cookies moisture content is significantly higher compared to the BC sample, mainly because of fiber BSG composition, which leads to better water absorption [34]. On the other hand, the physical fat entrapment during mixing, thanks to the newly formed network and the strong interaction between fat and BSG protein content, could lead to better fat absorption [35], and therefore to a higher moisture sample content. Moreover, BSG fiber plays an important role in water absorption, due to the greater number of hydroxyl groups, which allows more water interaction through hydrogen bonding [36].

The increased moisture of final products samples with increased BSG addition was also recently emphasized by Odeseye et al. [35], who showed that a moisture content smaller than 12% is able to inhibit enzymatic and microbial activities, improving the stability and shelf life of the biscuits.

The highest content of protein was identified in B5 sample (8.89%), while the BC sample registered a value of 6.65%. This result could be attributed to BSG higher protein content compared to the WF. Similar patterns of biscuits and cookies protein enrichment through the utilization of BSG were reported by Guo et al., Odeseye et al. and Öztürk et al. [28,35,37]. With respect to crude fiber content, addition of BSG increased the total crude fiber amount compared to that of BC samples, mainly due to BSG high content in crude fiber (ranging between 14.28% to 15.02%, Table 1). These results aligned with previous studies, which reported a linear increasement of cookies dietary fiber and baked snacks total fiber content, with increasing BSG levels [28,32]. In this light, Odeseye et al. [35] showed that the BSG biscuits fiber increment was attributed mainly to cellulose, lignin and hemicellulose BSG content, but also to the transition of insoluble fiber to soluble dietary fiber, owing to their alteration and formation of resistant starch.

The addition of BSG decreased total carbohydrate samples content (Table 5) compared to the BC sample. A possible explanation of decreasing carbohydrate BSG samples amount is mainly because of BSG’s low starch content (42.32%), considering that during the mashing process, the majority of starches were solubilized and digested [35]. Furthermore, the cookies energy (kcal/1005) decreased through BSG addition, mainly because of its lower carbohydrate content (42.12–43.77%, Table 1) compared to WF (74.78%, Table 1).

The total phenols higher values of baked samples compared to the BC sample could be explained by BSG’s higher total phenols content (211–258 mg GAE/100 g f.w., Table 1) compared to those of WF (31.14 mg GAE/100 g f.w. Table 1). This increment could be justified by BSG content in hydroxycinnamic acids such as p-coumaric, ferulic, sinapic and caffeic acids [38], as well as through its content in p-hydroxybenzoic, protocatechuic and chlorogenic acids, together with ferulic, p-coumaric and sinapic acids [21]. Ferulic and p-coumaric acids are identified in bigger amounts in BSG (35–490 mg/100 g d.w and 6.7–180 mg/100 g d.w., respectively), with ferulic acid being considered as the BSG target product [39]. Moreover, ferulic acid has similar effect to vitamin C, being used in prevention of food oxidation [39]. With respect to samples with different types of BSG addition, the highest total phenols amount was reached by the B4 sample, followed by B3 and B1 sample, respectively (Table 5). A strong Pearson relationship (r^2^ = 0.9965) was identified between BSG raw material and their corresponding final baked goods.

The phenolic molecules have been claimed by literature as having high antioxidant activity, exhibiting a potential protective role against chronic diseases such as diabetes, cancer and cardiovascular diseases [6,7]. Furthermore, hydroxycinnamic acids revealed anti-inflammatory, anti-platelet aggregation and antibacterial effects [40], and ferulic acid was claimed to have a positive influence on the gastrointestinal tract, being able to mediate a prebiotic modulation in gut microbiota [29].

In the present study, the BSG cookies antioxidant activity (Table 5) is bigger than the BC sample (Table 5), mainly due to higher BSG radical scavenging activity (22.56–25.41%, Table 2), compared to those of WF (7.98%, Table 1). A strong Pearson correlation (r^2^ = 0.9717) between BSG and final baked goods antioxidant activity emphasized the influence exhibited by the different types of BSG. The increased level of total phenols by the addition of BSG was emphasized also by Shih et al. [18] who showed that muffins with 20% BSG exhibited higher values of total phenols and antioxidant activity compared to the control sample. BSG total phenols could act as antioxidants in food manufacturing and processing, enhancing the antioxidant activity of the final baked products [18].

### 2.6. Mineral Samples Content

The cookies’ mineral content is displayed in Table 6. The addition of different BSG types influence in a positive way the macro and mineral content of the baked goods, excepting Na content, which was bigger in the BC sample. This could be justified by the higher amount of Na in WF (1284.05 mg/kg, Table 2) compared to BSG samples (486.14–514.37 mg/kg). Generally, BC sample is poorer in minerals (Table 6) compared to BSG samples, mainly due to less wheat flour minerals content (Table 3). In view of this, it is important to mention that, as reported by Gómez et al. [41], refined wheat flour, mainly used for bakery and pastry products, is a poor source in minerals, as follows: Ca (150 mg/kg), Mg (220 mg/kg), P (1080 mg/kg), Fe (11.70 mg/kg).

From the macro-minerals group, the richest sample in Ca and Mg was B3, registering values of 320 mg/kg and 159.49 mg/kg, respectively (Table 6). Microminerals such as Fe registered a higher value in B3 sample, while P was significantly higher in B5 sample (952.25 mg/kg). Pearson correlations of r^2^ = 0.998 and r^2^ = 0.997 showed a strong relationship between the total amount of BSG macro and micro-minerals compared with the final baked products.

Minerals such as Ca and Mg are reported by the literature as being involved in human metabolism by having a positive influence on bone structure, water and salt balance, while Zn deficiency could lead to dry skin, brittle nails and respiratory tract infections [42]. Furthermore, in the USA, the fortification of cereals goods in Ca is considered a positive factor in reducing the apparition of cancer colon and osteoporosis. A diet supplemented in Mg is highly recommended, since this could offer protection against obesity and diabetes mellitus type 2 [30].

### 2.7. Volatile Aroma Compounds Analysis

The aroma volatile profile of the final baked products and their odor perception are displayed in Table 7, and divided into six groups as follows: alcohols, aldehydes, ketones, esters, terpene and terpenoids and other compound classes. Positive Pearson correlations were identified between alcohols, aldehydes and ketones groups (r^2^ = 0.801, 0.798, 0.732) from BSG samples and final baked goods. The predominant volatile aroma compounds are aldehydes, with a total amount of 64.39% for the control sample and 68.31% to 75.37% for the BSG final baked samples. From the aldehydes group, the main compounds from BC sample were hexanal (32.03%), followed by 2-methyl-propanal (22.14%), while acetic acid, hexyl ester (10.81%) and 2-heptanone (8.6%) were the main representants of the esters and ketones group.

**Figure 3:**
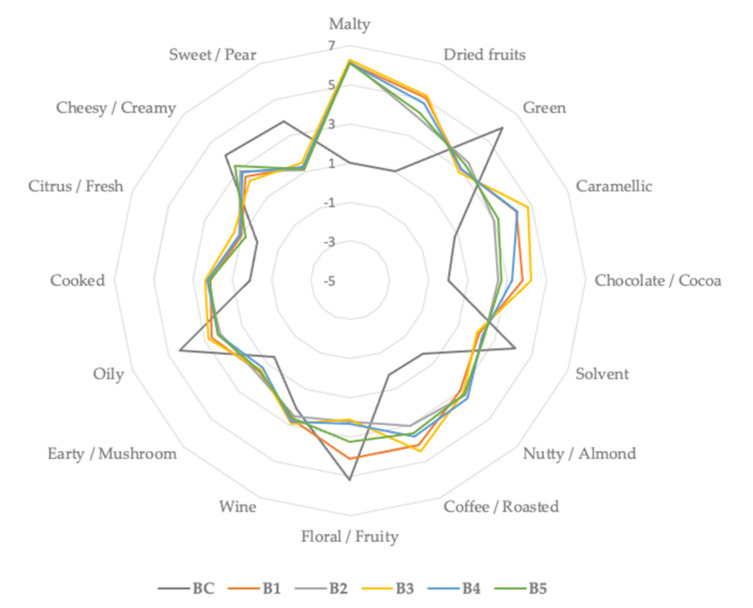
Aroma profile of control sample (BC) and samples with different BSG (brewers spent grains) types of main compounds classes identified during GC-MS analysis.

With respect to cookies samples with different BSG type addition, from the aldehydes, group 2-methyl-propanal reached the highest content in the B1 sample (25.03%), followed by hexanal, which was identified in a total amount of 20.29% in the B2 sample, by 3-methyl-butanal, mainly identified in the B3 sample and 2-methyl-butanal, mainly identified in the B1 sample (15.8%). The aforementioned compounds from the aldehydes group are responsible for odor perception such as malty, fruity, dry fruits, nutty, chocolate, cocoa, fatty and caramelly, leading to a pleasant consumer odor perception, and have been also identified in bigger amount in BSG samples (Table 4 and Figure 3). This finding suggests that the different BSG types exhibited a positive influence of the final baked aroma volatile compounds samples. For a better comprehension of the cookie’s aroma profile, samples were grouped considering their odor perception and illustrated in Figure 3.

The results are in line with those obtained by Fărcaș et al. [34], who mentioned almost the same aldehydes compounds in final baked goods such as bread, by replacing wheat flour with a different BSG addition. In snacks manufacturing, Ktenioudaki et al. [32] identified as main BSG aldehydes compounds 3-methyl-butanal, 2-methyl-butanal, pentanal and butanal, showing that the source of 2-methyl-butanal and 3-methyl-butanal might be through fermentation and the Maillard reaction, while 2-methyl-propanal could be exhibited through fermentation and dough processing.

The Maillard reaction is defined as a non-enzymatic process based on the reaction of amino-acids and sugars, which leads to the formation of melanoidins (brown pigments) and various volatile compounds such as aldehydes, amino-ketones, pyrazines, pyrroles, oxazole’s and thiophenes [43]. There are two main stages during the Maillard reaction: Strecker degradation and Ehrlich pathway. It seems that during Strecker degradation, thanks to the reaction between amino-acids and dehydroreductones, aldehydes are formed. For instance, 3-methyl-butanal was corelated to leucine amino-acids, 2-methyl-butanal to isoleucine and 2-methyl-propanal with valine amino-acid [43]. The formation of aldehydes, thanks to Strecker degradation, is sustainable considering that BSG is a rich source in amino-acids such as leucine (6.12% total protein), isoleucine (4.64% total protein) and valine (4.61% total protein), according to [9].

Hexanal (from the aldehydes group) is a result of lipid oxidation process, and is was reported by Bernal et al. as an off-flavor compound [44]. Hexanal was identified in a larger amount in control cookies samples (32.03%), while in BSG cookies the amount ranged between 16.18% to 20.29%. The higher hexanal amount in the BCM sample could be explained by the fact that WF is abundant in hexanal (37.05%, Table 3), while BSG hexanal content was identified in a range of 12.06% to 19.01%.

From the alcohols group, two compounds were identified (1-Pentanol, 1-Octen-3-ol), which according to Fărcaș et al. [23], are considered as lipid oxidation products with a bready, balsamic, green and earthy odor. 2-Heptanone and acetophenone were the identified compounds from ketones group, responsible for odor perception such as sweet fruity, fresh ripe and slightly green.

### 2.8. Color Parameters and Physical Characteristics of Cookies

The color of the cookies changed in a significant way by BSG addition (Figure 1), with L*, a* and b* values being significantly different (*p* < 0.05) from the control sample, as displayed in Table 8. The control sample had the highest values for L* and b*, while a* value was the lowest one. In the same vein, Heredia-Sandoval et al. [29] highlighted that L* and a* values were negatively influenced by the increased BSG levels, while b* value recorded a positive trend.

Likewise, Amoriello et al. [6] stated that BSG addition changed in a significant way the color intensity of the final baked products such as pizza, breadsticks and bread by decreasing lightness and yellowness (b*) parameters, while redness (a*) parameter registered increased levels. This could be mainly justified by BSG’s high amino-acid content, which favored the Maillard reaction and influenced the lightness and redness parameters [6]. In this vein, BSG is a rich in histidine (26.27% total protein), glutamic acid (16.59% total protein), aspartic acid (4.81% total protein), valine (4.62% total protein), arginine (4.51% total protein) but also in essential amino-acids such as lysine (14.31% total protein), leucine (6.12% total protein) and phenylalanine (4.64% total protein) [45], being able to facilitate Maillard reaction.

The main physical evaluation of cookies is considered as width (diameter) and thickness [29]. Moreover, Man et al. [46] mentioned spread ratio and weight as other physical characteristics that should be analyzed, together with color [29]. The physical cookies characteristics are displayed in Table 7. Regarding weight, there was a significant difference between control samples and BSG cookie (*p* < 0.05) mainly due to BSG higher content of protein and fibre, which better retained water during thermal treatment. Diameter did not differ in a significant way, while spread ratio encountered significant differences (*p* < 0.05) between the control sample and BSG samples (Table 7). Our results are in line with those reported by Guo et al. [40], who mentioned no significant differences on biscuits diameter, while thickness value registered only slightly variation with increasing BSG levels. With respect to spread ratio, significant differences were recorded between control samples and BSG cookies, and could be explained by the higher protein content of BSG compared to WF, which during the mixing and baking process is able to form a protein network able to enhance spread ratio values [8]. Moreover, Naibaho et al. [8] mentioned that higher BSG addition could be involved in reducing diameter values, and therefore influence the spread ratio value in a positive way.

### 2.9. Sensory Analysis

Combest et al. [47] highlighted that BSG addition could influence the sensorial features of the final baked goods such as odor, color, texture and flavor, and therefore the consumers’ sensory analysis regarding the acceptance of the new developed products is highly required. The use of BSG in food manufacturing was associated with a positive effect in increasing food’s appeal, consumers placing sensory properties after health issues [8,48].

In the present study, for the appearance attribute significant differences were encountered between control sample and B3 sample (*p* < 0.05). This could indicate that panelists were able to visually discriminate the sample based on their color and final surface cookies degree imperfections. The highest appearance score was registered by the B3 sample, followed by B4 and B1 (Table 9). It was stated that BSG used in food manufacturing is expected to increase food appeals, based on consumers’ awareness to place health food benefits as a main criterion in food selection. Most of the published researchers suggested that an addition of BSG as a substitution ingredient between 20–25% is considered to be effective from the nutritional point of view [8]. For instance, the phrase “high in fiber” is associated by consumers with outcomes such as “better digestion”, “satiety”, “natural and pure” and “weight control” [48]. Moreover, Naibaho et al. [8] stated that the addition of BSB had changed the color of the final baked goods into a unique dark color, which is assumed to be healthier. The color changes could be also a result of pigment degradation, caramelization, Maillard reaction and hydrolysis.

Texture was analyzed from attributes such as hardness, chewiness and crispiness (Table 9). Crispiness was defined as the force and noise produced, while the sample breaks during the chewing sample on the molar teeth [49]. With respect to hardness and chewiness, addition of BSG increased the samples’ score, but to levels which were appreciated by consumers (Table 8). The increased hardness values in BSG cookies compared to BC could be explained by pentopans, a fiber BSG component that might cause hardening through gluten protein cross linking [36]. In the same vein, Petrovic et al. [15] showed a linear positive hardness and chewiness increasement on cookies, as the BSG level increased due to its high content in proteins and fiber.

With respect to taste and aroma attribute, the B3 sample registered the highest hedonic score (8.75), followed by B4 and B5 samples, respectively (Table 9). The aroma of the final baked products could be influenced by the volatile compounds identified previously (Table 7), such as aldehydes group from which 2-methyl-butanal, 2-methyl-propanal, 3-methyl-butanal and hexanal reached higher values and odor perception, such as fruity, chocolate, coffee, malty, nutty and rummy, characterized the aforementioned aroma volatile compounds; this could also explain the consumers’ preferences towards the B3 sample. In the same idea, a study realized by Combest et al. [47] with 37 college students as panelists emphasized that BSG presented as a raw material in mason jars was characterized as having odor perceptions such as “sweet”, “warm”, “earthy” and “nutty”. Moreover, BSG aroma snacks was related to the fresh aroma of homemade bread, characterized as smelling “like home”.

The overall appreciation attribute encountered that the B3 sample was highly appreciated by consumers with a hedonic score of 8.97, followed by the B4 and B1 samples (Table 8).

## 3. Materials and Methods

### 3.1. Materials

The raw materials such as flour, butter, eggs, sugar, baking powder and salt were purchased from a Cluj-Napoca store, Romania. The wheat flour (WF) was achieved from a mill (Boromir, Deva, Romania) with an ash content of 0.48% (according to Romanian classification, [50]) and a wet gluten content of 29.57%, as previously mentioned by Man et al., 2019, [49]. The five BSG types were kindly offered by a local craft brewery from Cluj-Napoca, dried during 12 h in a professional dehydrator (Hendi Profi Line, Utrecht, the Netherlands) at 60 °C, and grinded with a laboratory professional mill (IKA A10, Staufen, Germany). A sieve of 0.8 mm was used to sieved the grinded BSG, aiming to have the same uniformity in particles. The microbiological safety of the BSG ingredients were evaluated through SR ISO 21527-2/2008 standard [51], according to the method described by Chiș et al. [52] and to Romanian Regulations (Order no. 27/2011), [53].

### 3.2. Types of Malt Used in Obtaining Brewers Spent Grain (BSG)

Table 10 displayed the types of malt (%) used for BSG by-product, the malt kilning temperature and samples codification, as follows:

### 3.3. Cookie’s Manufacturing

Five blends with the same BSG addition (20%, 80:20 WF:BSG, w:w) to the wheat flour content were made (Table 11). The addition of replacing 20% of wheat flour with BSG was based on our previously published articles [34], but also on an extensive literature research [8,15,29,54]. After this, butter (65% fat) and powdered sugar were mixed until a cream base was obtained. WF, BSG, salt and baking powder were added by mixing all the ingredients with an automatic mixer (KitchenAid^®^ Precise Heat Mixing Bowl, Greenville, OH, USA). Technological parameters such as mixing, resting and baking were made according to [49] and displayed in Table 11. A control sample was manufactured in the same conditions with 100% WF. All the samples were laminated until reaching a thickness of 0.8 cm by using a laminator (Flamic SF600, Vicenza, Italy) and shaped with a 5.5 cm cylindrical shape diameter. The baking process was realized in an electric oven (Zanolli, Verona, Italy), and the samples were cooled to room temperature and packed in polypropylene bags until further analysis. The safety of the final baked goods were evaluated according to SR ISO 21527-2/2008 standard [51], as mentioned by Chiș et al. [52] and to Romanian Regulations (Order no. 27/2011) [53].

### 3.4. Proximate Composition Analysis

The proximate composition such as moisture, protein, lipids, ash and crude fiber were analyzed according to AACC (2000)-approved methods [55], such as AACC 44-15.02, AACC 46-11.02 (total nitrogen multiplied with 5.7 protein conversion factor), AACC 30-25.01, AACC 08-01.01 and AACC 32-07.01, respectively. Carbohydrates were calculated as the difference between 100 and moisture, ash, proteins, lipids and crude fiber percentages, as showed by Man et al. [49].

### 3.5. Physical Evaluation of Cookies and Color

Weight, thickness, diameter and spread ratio were measured as described by Man et al. [49], and color was performed based on the method mentioned by Heredia-Sandoval et al. [29]. The weight was measured on an analytical balance, and thickness and diameter were measured using a Vernier calliper (Mitutuyo, Kawasaki, Japan) with an accuracy of 0.01 mm. An NH 300 portable colorimeter (Shenzhen Threenh Technology Co., Ltd, Shenzhen, China) on the basis of CIE L* (luminosity), a* (red/green coordinate), b* (yellow/blue coordinate) color system was used. The colorimeter was previously calibrated through its auto black and white calibration system, and all measurements were repeated three times and presented as means ± sd.

### 3.6. Total Phenolic Contents

The methanolic extracts were obtained by triplicate extraction of 2 g of each dried BSG samples, with 60 mL of acidified methanol (85:15 *v/v*, MeOH:HCl) for 30 min using an ultrasonic bath (SONOREX™ SUPER RK 100 H, Bandelin, Germany) and then were centrifuged at 9000 rpm for 10 min (centrifuge with cooling, model Universal 320R, Hettich). After filtration under reduced pressure, the total extracts were evaporated in a rotary evaporator (Heidolph Instruments GmbH& Co. KG, Schwabach, Germany) and recovered in 10 mL methanol. Millipore filters (Millipore, Merck KGaA, Darmstadt, Germany) were used for sample filtering. Total phenolic content was determined through Folin-Ciocalteau method, as described by Chiș et al. [56]. Briefly, 100 µL methanolic extract was mixed with 500 µL Folin Ciocalteu reagent. After this, 6 mL of distilled water and 2 mL of 7.5% Na_2_CO_3_ were added and mixed, and distilled water was used to brought up the obtained solution to 10 mL. A spectrophotometer (Shimadzu Corporation, Kyoto, Japan) at a wavelength of 750 nm was used for absorbance measurement. The results were made in triplicate, and expressed as mg of gallic acid equivalent (GAE)/100 g fresh weight (y = 0.88340x − 0.0245; r^2^ = 0.9989).

### 3.7. Antioxidant Activity

The antioxidant activity was measured spectrophotometrically by using DPPH (2,2-diphenyl-1-picrylhydrazyl) method, as described by Fărcaș et al. [23]. Shortly, the methanolic extracts (35 µL of sample) were mixed with 250 µL DPPH solution (80 µM) and measured at 515 nm, by using a microplate BioTek Synergy HT reader (BioTek Instruments, Winooski, VT, USA). The radical scavenging activity was calculated as a difference between DPPH absorbance and the sample absorbance, dived by DPPH absorbance and multiplied by 100.

### 3.8. Minerals’ Determination

Minerals were analyzed as described in our recent study [57]. The samples were briefly dried at 105 °C for 4 h, then cooled down in an exicator and grinded using a laboratory professional mill (IKA A10, Staufen, Germany) to a size particle samples dimension of 200 µm. For the mineralization step, a Bergdorf MWS-2 digester (Bergdorf, Achalm, Germany) was used, and 1 g of sample was mixed with 15 mL HNO3 and 6 mL H_2_O_2_. The mineralization step was realized 120 min, at a temperature of 120 °C. Optima 5300DV (PerkinElmer, Waltham, MA, USA) spectrometer coupled with a CETAC 6000AT+ (CETAC, Omaha, NE, USA) ultrasonic nebulizer, and an inductive plasma optical emission spectrometry (ICP-OES) tool was used for further analysis. The sample uptake rate was 1.5 mL/min, 15 L/min plasma flow, 2.0 L/min and 0.8 L/min for auxiliary and nebulizer low, respectively. Multi-elemental solutions of 1000 mg/L ICP Standard Certipur^®^ (Merck, Darmstadt, Germany) were used for calibration. All samples were analyzed in triplicate.

### 3.9. Volatile Compounds

Aroma volatile compounds determination was performed on ITEX (in-tube extraction technique), followed by GC-MS (gas chromatography-mass spectrometry) separation and identification of compounds through a GC-MS QP-2010 equipment (Shimadzu Scientific Instruments, Kyoto, Japan), as described in our publications [34,58]. A capillary column (ZB-5 ms, 30 m × 0.25 mm i.d. × 0.25 µm, Phenomenex, Torrance, CA, USA) and a Combi-PAL AOC-5000 autosampler (CTC Analytics, Zwingen, Switzerland) were used. 2 g of each sample were briefly incubated in a sealed headspace vial at a temperature of 60 °C, for 10 min. A porous polymer fiber microtrap (ITEX-2TRAPTXTA, Tenax TA 80/100 mesh, ea) was carried out for the adsorption (15 strokes) of the volatile compounds and their thermal desorption was performed by a CombiPAL AOC-5000 autosampler. The parameters of the column were as follows: helium as a carrier gas, time and temperature ranged as follows: 5 min at 30 °C, rose to 110 °C at 4 °C/min and heated at 250 °C at 20 °C/min and held for 5 min with a mass range scanned between 35–350 m/z. NIST27 and NIST147 mass spectra libraries and databases such as www.pherobase.com (accessed on 7 October 2021) [59] or www.flavornet.org (accessed on 7 October 2021) [60] were used for the identification of the volatile compounds. The samples were analyzed in triplicate and the results were expressed as arbitrary units (a.u.) from the relative peak areas (one a.u. corresponding to 10.000 units of peak area).

### 3.10. Sensory Analysis

The sensory evaluation was done using 75 volunteers, staff and students from the Faculty of Food Science and Technology between the ages of 18 and 65 years old (age 18–35: 60%, age 36–65: 40%)/(64% female, 36% male). The panelists were selected based on their regular cookies’ consumption, availability and their interest. Six samples of freshly made (within 4 h) cookies were evaluated in each session under normal daylight conditions. All samples were anonymously coded, and two replications of each sample were presented to panelists. They were asked to evaluate cookies’ visual appearance, hardness, crispiness, chewiness, taste and aroma and overall appreciation using a 9-point hedonic scale ranging from 9 (like extremely) to 1 (dislike extremely), as described by Chiș et al. [42] and Man et al. [46]. Water was provided to rinse the mouth between evaluations.

### 3.11. Statistical Analysis

Duncan multiple comparation test (*p* < 0.05) with SPSS version 19 software (IBM Corp., Armonk, NY, USA) was used for statistical analysis. All samples were analyzed in three replicates (*n* = 3) and expressed as means ± sd. Different superscript small letters in a row indicates significant differences between samples (*p* < 0.05). Pearson correlation was used to better emphasize the correlation between raw materials and final baked goods, performed on Minitab 19.1 (Minitab Inc., State College, PA, USA), with a 95% confidence level.

## 4. Conclusions

Five different types of BSG by-products with an addition of 20% were used in cookies manufacturing, replacing wheat flour. The substitution of wheat flour with these unconventional ingredients enhanced the nutritional values of the final baked goods (increased protein, fiber, lipids, minerals, total phenols, antioxidant activity) and improved their sensorial attributes. BSG aroma volatile compounds positively influenced the final cookies’ aromatic profile, leading to pleasant odor perceptions. With respect to the texture parameters, hardness, crispiness and chewiness reached significant positive hedonic value (*p* < 0.05) scores compared to the control sample, emphasizing that BSG-enriched samples were also appreciated by consumers from the textural point of view. Furthermore, the products manufactured with BSG from light and dark malt mixture was the most appreciated sample by panelists, mainly because of its intense color, texture parameters and special aromatic profile.

Although the recovery of residues and agri-food by-products is already considered a research topic of great interest, studies similar to the proposed article are extremely important, as they have a major contribution in creating a scientific database to support the sustainability of reintegration processes and to promote the concept of circular bioeconomy.

## Figures and Tables

**Table 1:** Proximate composition of dried BGS (brewers spent grains) samples and WF (wheat flour).

Parameters	WF	BSG1	BSG2	BSG3	BSG4	BSG5
Moisture (%)	14.5 ± 0.04 ^b^	7.21 ± 0.05 ^a^	7.52 ± 0.22 ^a^	7.49 ± 0.03 ^a^	7.51 ± 0.45 ^a^	7.35 ± 0.05 ^a^
Protein (%)	10.03 ± 0.25 ^a^	24.90 ± 0.03 ^bc^	25.02 ± 0.12 ^bc^	24.42 ± 0.31 ^bc^	24.00 ± 0.34 ^b^	25.80 ± 0.09 ^c^
Lipids (%)	1.01 ± 0.03 ^a^	6.49 ± 0.04 ^bc^	7.05 ± 0.05 ^cd^	5.96 ± 0.21 ^b^	6.60 ± 0.32 ^bc^	6.90 ± 0.06 ^c^
Ash (%)	0.48 ± 0.02 ^a^	3.46 ± 0.33 ^bc^	2.85 ± 0.08 ^b^	3.82 ± 0.76 ^c^	3.12 ± 0.04 ^bc^	3.01 ± 0.04 ^bc^
Crude fiber (%)	0.37 ± 0.44 ^a^	14.28 ± 0.29 ^b^	14.99 ± 0.34 ^bc^	14.54 ± 0.18 ^b^	15.02 ± 0.51 ^bc^	14.82 ± 0.55 ^bc^
Total carbohydrates (%)	74.78 ± 0.78 ^c^	43.66 ± 0.70 ^b^	42.57 ± 0.77 ^a^	43.77 ± 1.33 ^b^	43.75 ± 1.62 ^b^	42.12 ± 1.1.5 ^a^
Total phenols (mg GAE/100 g f.w)	31.14 ± 0.09 ^a^	236.08 ± 0.37 ^d^	211.26 ± 0.55 ^b^	258.12 ± 0.76 ^f^	242.56 ± 0.45 ^e^	220.16 ± 0.22 ^c^
Antioxidant activity (%)	7.98 ± 0.55 ^a^	24.31 ± 0.23 ^c^	22.56 ± 0.89 ^b^	26.77 ± 0.33 ^d^	24.59 ± 0.55 ^c^	22.88 ± 0.44 ^b^

Different small letter in a row indicates the significant difference between samples (*p* < 0.05); values are presented as means of three determinations ± SD; f.w.—fresh weight.

**Table 2:** Color characteristics (CIE L*, a*, b* values) of BSG (brewers spent grains) types and WF (wheat flour).

Samples	L*	a*	b*
WF	98.84 ± 0.77 ^f^	1.38 ± 0.32 ^a^	11.03 ± 0.57 ^a^
BSG1	52.46 ± 0.95 ^b^	10.24 ± 0.19 ^c^	21.75 ± 0.030 ^c^
BSG2	60.36 ± 0.63 ^d^	7.42 ± 0.10 ^b^	20.19 ± 0.53 ^b^
BSG3	48.24 ± 0.81 ^a^	8.97 ± 0.23 ^bc^	21.76 ± 0.30 ^c^
BSG4	59.61 ± 0.77 ^c^	9.53 ± 0.14 ^bc^	22.38 ± 0.34 ^c^
BSG5	61.96 ± 0.56 ^e^	9.32 ± 0.18 ^bc^	22.71 ± 0.34 ^d^

Different superscript letters in a column indicates significant differences between samples; lightness (L*), redness (a*), yellowness (b*).

**Table 3:** Mineral’s content of dried BSG (brewers spent grains) and WF (wheat flour) samples.

Parameters (mg/kg)	WF	BSG1	BSG2	BSG3	BSG4	BSG5
**Macrominerals**						
Calcium	188.12 ± 0.73 ^a^	1241.40 ± 0.67 ^c^	1232.07 ± 0.62 ^b^	1271.11 ± 0.23 ^f^	1258.37 ± 0.52 ^e^	1244.13 ± 0.22 ^d^
Potassium	1284.05 ± 0.89 ^f^	486.14 ± 0.55 ^a^	494.81 ± 0.43 ^b^	508.03 ± 0.13 ^d^	514.37 ± 0.76 ^e^	503.15± 0.18 ^c^
Sodium	76.10 ± 0.67 ^a^	218.30 ± 0.34 ^d^	203.38 ± 0.21 ^b^	226.35 ± 0.39 ^e^	225.45 ± 0.32 ^e^	211.68 ± 0.65 ^c^
Magnesium	201.32 ± 0.55 ^a^	741.21 ± 0.54 ^d^	752.87 ± 0.16 ^e^	761.14 ± 0.55 ^f^	729.35 ± 0.44 ^b^	738.98 ± 0.54 ^c^
Total	1561.47 ± 2.84 ^a^	2687.05 ± 2.10 ^c^	2683.13 ± 1.42 ^b^	2766.63 ± 1.30 ^f^	2727.54 ± 2.04 ^e^	2697.94 ± 1.59 ^d^
**Microminerals**						
Iron	9.12 ± 0.49 ^a^	123.46 ± 0.33 ^d^	104.78 ± 0.32 ^b^	131.24 ± 0.33 ^f^	126.86 ± 0.62 ^e^	116.06 ± 0.33 ^c^
Zinc	8.41 ± 0.38 ^a^	141.08 ± 0.17 ^d^	129.04 ± 0.12 ^b^	144.17 ± 0.22 ^e^	133.16 ± 0.45 ^c^	129.14 ± 0.56 ^b^
Manganese	5.12 ± 0.73 ^a^	47.11 ± 0.41 ^d^	49.16 ± 0.19 ^e^	46.29 ± 0.11 ^d^	39.75 ± 0.17 ^b^	41.80 ± 0.13 ^c^
Copper	6.22 ± 0.32 ^a^	16.08 ± 0.23 ^cd^	17.12 ± 0.56 ^d^	15.06 ± 0.20 ^bc^	14.81 ± 0.55 ^b^	13.98 ± 0.38 ^b^
Phosphorus	1027.23 ± 0.88 ^a^	3003.92 ± 0.31 ^d^	3011.54 ± 0.43 ^e^	2995.38 ±0.42 ^c^	2989.73 ± 0.49 ^b^	3034.13 ± 0.45 ^f^
Total	1056.10 ± 2.80 ^a^	3331.65 ± 1.45 ^d^	3311.64 ± 1.62 ^c^	3332.14 ± 1.28 ^d^	3304.31 ± 2.28 ^b^	3335.11 ± 1.85 ^e^

Different small letter in a row indicates the significant difference between samples (*p* < 0.05); values are presented as means of three determinations ± SD.

**Table 4:** The volatile fingerprints of WF (wheat flour) and dried BSG (brewers spent grains) samples expressed as arbitrary units (a.u.) from the relative peak areas.

Volatile Compounds	RT (min)	WF	BSG1	BSG2	BSG3	BSG4	BSG5	Odour Perception
***Alcohols***								
1-pentanol	5.236	n.d.	2.72 ± 0.02 ^a^	3.65 ± 0.34 ^b^	4.63 ± 0.12 ^bc^	4.09 ± 0.56 ^bc^	5.36 ± 0.23 ^c^	Pungent. Fermented, bready, wine, cereal, balsamic, fruity
1-octen-3-ol	14.683	n.d.	0.52 ± 0.12 ^bc^	0.53 ± 0.22 ^bc^	0.45 ± 0.35 ^a^	0.63 ± 0.21 ^c^	0.47 ± 0.45 ^b^	Mushroom, earthy, green, oily, umami sensation
2-ethyl-1-hexanol	17.046	n.d.	n.d.	0.08 ± 0.02 ^ab^	n.d.	0.03 ± 0.01 ^a^	0.09 ± 0.02 ^ab^	Citrus fresh, floral, oily, sweet
Total		n.d.	3.24 ± 0.15 ^a^	4.26 ± 0.58 ^b^	5.08 ± 0.47 ^d^	4.75 ± 0.78 ^c^	5.92 ± 0.70 ^e^	
***Aldehydes***								
2-methylpropanal	2.512	52.1 ± 0.77 ^f^	22.8 ± 0.04 ^b^	29.8 ± 0.23 ^e^	19.14 ± 0.14 ^a^	24.11 ± 0.21 ^c^	27.17 ± 0.13 ^d^	Wine, solvent, malty, fruity
3-methylbutanal	3.337	n.d.	27.0 ± 0.78 ^bc^	22.34 ± 0.87 ^a^	29.05 ± 0.39 c	28.0 ± 0.81 ^c^	25.13 ± 0.35 ^b^	Dried fruits, nutty, chocolate, cocoa, fatty
2-methylbutanal	3.469	n.d.	23.0 ± 0.56 ^d^	11.8 ± 0.73 ^a^	24.19 ± 0.82 ^e^	17.3 ± 0.85 ^c^	15.03 ± 0.22 ^b^	Malty, cocoa, chocolate, coffee, caramellic, malty, nutty, rummy
hexanal	6.468	37.05 ± 0.88 ^e^	12.06 ± 0.44 ^a^	19.81 ± 0.55 ^d^	14.20 ± 0.83 ^b^	15.16 ± 0.29 ^bc^	17.16 ± 0.42 ^d^	Intense green, aldehydic, fruity, oily
heptanal	11.036	n.d.	0.7 ± 0.02 ^d^	0.29 ± 0.01 ^a^	0.36 ± 0.02 ^ab^	0.46 ± 0.02 ^bc^	0.58 ± 0.02 ^cd^	Green, oily, fatty, cognac, almond
2-heptenal	13.613	n.d.	n.d.	0.09 ± 0.01 ^ab^	0.09 ± 0.01 ^ab^	n.d.	0.04 ± 0.01 ^a^	Intense green, fatty, oily, fruity
octanal	15.74	n.d.	0.27 ± 0.02 ^c^	0.2 ± 0.01 ^b^	0.21 ± 0.01 ^b^	0.11 ± 0.01 ^a^	0.12 ± 0.02 ^a^	Waxy, fat, soap, lemon, green
nonanal	20.151	n.d.	0.17 ± 0.02 ^ab^	0.19 ± 0.03 ^ab^	0.10 ± 0.01 ^a^	n.d.	n.d.	Waxy, fat, citrus, green
2-butyl-2-octenal	26.845	2.1 ± 0.02 ^b^	0.08 ± 0.01 ^a^	0.12 ± 0.02 ^a^	0.06 ± 0.02 ^a^	0.09 ± 0.02 ^a^	0.17 ± 0.02 ^a^	Green, vegetable, herbal
Total		91.25 ± 0.90 ^e^	86.08 ± 1.89 ^c^	84.64 ± 2.46 ^a^	87.40 ± 2.25 ^d^	85.23 ± 2.21 ^b^	85.40 ± 1.19 ^b^	
***Cetones***								
2-hexanone	5.997	n.d.	0.1 ± 0.01 ^ab^	n.d.	n.d.	0.08 ± 0.01 ^ab^	0.02 ± 0.01 ^a^	Fruity, fungal, meaty, buttery
2-heptanone	10.455	n.d.	0.82 ± 0.02 ^a^	1.09 ± 0.03 ^c^	0.96 ± 0.04 ^b^	1.26 ± 0.05 ^d^	1.17 ± 0.06 ^cd^	Cheesy, fruity, spicy, creamy
6-methyl-5 hepten-2-one	12.382	n.d.	0.08 ± 0.02 ^a^	0.26 ± 0.02 ^ab^	n.d	n.d.	0.09 ± 0.01 ^a^	Fruity, green, citrus, herbal, creamy
3-Octen-2-one	17.267	n.d.	0.44 ± 0.21 ^b^	0.37 ± 0.07 ^a^	0.51 ± 0.02 ^c^	0.48 ± 0.03 ^bc^	0.63 ± 0.03 ^d^	Earthy, spicy, herbal, sweet, nutty, fermented
Acetophenone	18.402	8.12 ± 0.23 ^d^	0.16 ± 0.01 ^b^	0.19 ± 0.02 ^b^	0.08 ± 0.03 ^a^	0.56 ± 0.04 ^c^	n.d.	Floral, must, spicy, almond, nutty
3,5-Octadien-2-one	18.643	n.d.	0.24 ± 0.02 ^a^	0.35 ± 0.05 ^b^	0.44 ± 0.12 ^c^	0.31 ± 0.02 ^ab^	0.29 ± 0.02 ^ab^	Fruity, woody, mushroom, fatty
-1-[4-(2-hydroxypropan-2-yl)phenyl]ethanone	25.81	n.d.	1.6 ± 0.08 ^c^	1.04 ± 0.22 ^b^	0.40 ± 0.13 ^a^	1.12 ± 0.15 ^ab^	n.d.	Warm spicy, woody, herbaceous
Total		8.12 ± 0.23 ^d^	3.44 ± 0.37 ^b^	3.31 ± 0.41 ^b^	2.39 ± 0.34 ^a^	3.81 ± 0.30 ^c^	2.21 ± 0.11 ^a^	
***Terpene and Terpenoids***								
β -trans-Ocimene	12.356	n.d.	0.3 ± 0.01 ^b^	n.d.	0.36 ± 0.02 ^bc^	0.3 ± 0.02 b	0.09 ± 0.02 ^a^	Tropical fruits, woody, sweet, floral, green
β -Pinene	14.406	n.d.	0.98 ± 0.25 ^cd^	0.85 ± 0.31 ^c^	0.16 ± 0.02 ^ab^	0.22 ± 0.01 ^b^	0.11 ± 0.02 ^a^	Woody, green, pine
p-cymene	16.599	n.d.	1.64 ± 0.25 ^d^	0.92 ± 0.11 ^c^	0.38 ± 0.05 ^a^	0.59 ± 0.03 ^b^	0.68 ± 0.03 ^b^	Fresh citrus, terpenic, woody, spicy
D-Limonene	16.803	0.63 ± 0.02 ^a^	2.71 ± 0.03 ^c^	3.32 ± 0.05 ^d^	2.22 ± 0.12 ^b^	2.72 ± 0.45 ^c^	2.75 ± 0.31 ^c^	Citrus, fresh, sweet
Total		0.63 ± 0.02 ^a^	5.63 ± 0.54 ^e^	5.09 ± 0.47 ^d^	3.12 ± 0.21 ^b^	3.83 ± 0.51 ^c^	3.63 ± 0.38 ^b^	
***Others***								
methyl 2,4-dimethyl-3-oxopentanoate	14.17	n.d.	n.d.	n.d.	n.d.	0.11 ± 0.01 ^a^	0.11 ± 0.01 ^a^	Walnut, dry, woody
2-pentylfuran	15.085	n.d.	1.36 ± 0.04 ^a^	2.14 ± 0.05 ^c^	2.01 ± 0.71 ^c^	1.84 ± 0.37 ^b^	2.55 ± 0.45 ^d^	Green, earthy, beans, musty, cooked, caramellic
3,7-Dimethyl-1-octene	16.42	n.d.	0.25 ± 0.02 ^ab^	0.57 ± 0.02 ^bc^	n.d.	0.43 ± 0.02 ^b^	0.19 ± 0.02 ^a^	Woody, pine, herbal
Total		n.d.	1.61 ± 0.06 ^a^	2.71 ± 0.07 ^d^	2.01 ± 0.71 ^b^	2.38 ± 0.40 ^c^	2.85 ± 0.48 ^e^	

Different small letter in a row indicates the significant difference between samples (*p* < 0.05); values are presented as means of three determinations ± SD; n.d.—not detected.

**Table 5:** Proximate characteristics (% f.w.) of the cookie’s samples.

Parameters	BC	B1	B2	B3	B4	B5
Moisture (%)	7.04 ± 0.09 ^a^	7.88 ± 0.05 ^b^	8.61 ± 0.26 ^c^	8.21 ± 0.07 ^bc^	7.94 ± 0.37 ^b^	8.56 ± 0.24 ^c^
Protein (%)	6.65 ±0.43 ^a^	8.52 ± 0.21 ^b^	8.76 ± 0.3 ^bc^	8.64 ± 0.54 ^bc^	8.58 ± 0.11 ^b^	8.89 ± 0.03 ^bc^
Lipids (%)	18.12 ± 0.09 ^a^	18.86 ± 0.57 ^c^	18.93 ± 0.11 ^c^	18.64 ± 0.52 ^b^	18.73 ± 0.31 ^bc^	18.81 ± 0.08 ^bc^
Ash (%)	0.71 ±0.03 ^a^	1.14 ± 0.22 ^cd^	0.98 ± 0.03 ^b^	1.19 ± 0.23 ^d^	1.07 ± 0.03 ^bcd^	1.03 ± 0.02 ^bc^
Crude fiber (%)	0.69 ± 0.02 ^a^	3.22 ± 0.11 ^b^	3.36 ± 0.13 ^bc^	3.27 ± 0.05 ^b^	3.38 ± 0.12 ^bc^	3.33 ± 0.05 ^bc^
Total carbohydrates (%)	66.79 ± 0.66 ^b^	60.38 ± 1.16 ^a^	59.36 ± 0.83 ^a^	60.05 ± 1.41 ^a^	60.30 ± 0.94 ^a^	59.38 ± 0.42 ^a^
Energy (kcal/100 g)	477.54 ± 0.04 ^c^	464.32 ± 0.05 ^b^	461.54 ± 0.09 ^a^	461.39 ± 0.34 ^a^	463.02 ± 0.55 ^b^	461.08 ±0.77 ^a^
Total phenols (mg GAE/100 g f.w)	9.42 ± 0.03 ^a^	50.49 ± 0.20 ^c^	47.01 ± 0.02 ^b^	53.58 ± 0.05 ^d^	51.40 ± 0.41 ^c^	48.26 ± 0.17 ^b^
Antioxidant activity (%)	4.79 ± 0.09 ^a^	13.54 ± 0.03 ^b^	14.59 ± 0.01 ^bc^	17.45 ± 0.11 ^d^	15.25 ± 0.12 ^c^	13.73 ± 0.12 ^b^

Different small letter in a row indicates the significant difference between samples (*p* < 0.05); values are presented as means of three determinations ± SD; f.w.—fresh weight; BSG (brewers spent grains); BC—cookies sample manufactured with 100% WF (wheat flour); B1—cookies sample manufactured with 20% BSG1; B2—cookies sample manufactured with 20% BSG2; B3—cookies sample manufactured with 20% BSG3; B4—cookies sample manufactured with 20% BSG4; B5—cookies sample manufactured with 20% BSG5.

**Table 6:** The macro and microminerals content of developed cookies.

Parameters (mg/kg)	BC	B1	B2	B3	B4	B5
**Macrominerals**						
Calcium	222.75 ± 0.77 ^a^	317.54 ± 0.29 ^b^	316.70 ±0.81 ^b^	320.22 ± 0.55 ^d^	319.07 ± 0.22 ^cd^	317.79 ± 0.18 ^bc^
Potassium	773.50 ± 0.44 ^d^	701.69 ± 0.26 ^a^	702.47 ± 0.41 ^ab^	703.66 ± 0.19 ^bc^	704.23 ± 0.55 ^c^	703.22 ± 0.42 ^bc^
Sodium	253.31 ± 0.49 ^a^	265.99 ± 0.22 ^bc^	264.65 ± 0.64 ^b^	266.72 ± 0.92 ^c^	266.64 ± 0.18 ^c^	265.40 ± 0.1 ^bc^
Magnesium	109.11 ± 0.62 ^a^	157.70 ± 0.33 ^bc^	158.75 ± 0.37 ^cd^	159.49 ± 0.72 ^d^	156.63 ± 0.52 ^b^	157.50 ± 0.22 ^bc^
Total	1358.55 ± 2.32 ^a^	1442.92 ±1.10 ^b^	1442.57 ± 2.23 ^b^	1450.09 ± 2.38 ^d^	1446.57 ± 1.47 ^c^	1443.90 ± 0.96 ^b^
**Microminerals**						
Iron	6.79 ± 0.17 ^a^	17.08 ± 0.11 ^c^	15.40 ± 0.05 ^b^	17.78 ± 0.51 ^c^	17.39 ± 0.36 ^c^	16.42 ± 0.07 ^bc^
Zinc	5.59 ± 0.16 ^a^	17.53 ± 0.03 ^c^	16.44 ± 0.06 ^b^	17.81 ± 0.27 ^c^	16.82 ± 0.13 ^b^	16.45 ± 0.03 ^b^
Manganese	2.58 ± 0.73 ^a^	6.36 ± 0.31 ^bc^	6.55 ± 0.05 ^bc^	6.29 ± 0.04 ^bc^	5.70 ± 0.02 ^b^	5.88 ± 0.05 ^b^
Copper	2.27 ± 0.12 ^a^	4.65 ± 0.13 ^b^	4.75 ± 0.02 ^b^	4.56 ± 0.07 ^b^	4.54 ± 0.55 ^b^	4.46 ± 0.12 ^b^
Phosphorus	771.63 ± 0.53 ^a^	949.54 ± 0.90 ^bc^	950.22 ± 0.87 ^c^	948.77 ±0.78 ^b^	948.26 ± 0.49 ^b^	952.25 ± 0.51 ^d^
Total	788.87 ± 1.71 ^a^	995.16 ± 1.48 ^c^	993.36 ± 1.05 ^b^	995.21 ± 1.67 ^c^	992.70 ± 1.55 ^b^	995.47 ± 0.63 ^c^

Different small letter in a row indicates the significant difference between samples (*p* < 0.05); values are presented as means of three determinations ± SD; f.w.—fresh weight; BSG (brewers spent grains); BC—cookies sample manufactured with 100% WF (wheat flour); B1—cookies sample manufactured with 20% BSG1; B2—cookies sample manufactured with 20% BSG2; B3—cookies sample manufactured with 20% BSG3; B4—cookies sample manufactured with 20% BSG4; B5—cookies sample manufactured with 20% BSG5.

**Table 7:** Volatile aroma compounds of the final baked goods expressed as arbitrary units (a.u.) from the relative peak areas.

Volatile Compounds	RT (min)	B1	B2	B3	B4	B5	BM	Odor Perception
***Alcohols***								
1-pentanol	5.236	1.22 ± 0.03 ^b^	0.68 ± 0.21 ^a^	1.12 ± 0.07 ^b^	2.52 ± 0.13 ^c^	2.95 ± 0.23 ^c^	0.23 ± 0.02 ^a^	Pungent, fermented, bready, wine, cereal, balsamic, fruity
1-octen-3-ol	14.683	0.09 ± 0.01 ^a^	1.27 ± 0.11 ^b^	1.13 ± 0.21 ^b^	1.58 ± 0.03 ^b^	1.45 ± 0.22 ^b^	0.06 ± 0.01 ^a^	Mushroom, earthy, green, oily, umami sensation
Total		1.31 ± 0.04 ^ab^	1.95 ± 0.32 ^b^	2.25 ± 0.28 ^b^	4.10 ± 0.16 ^c^	4.40 ± 0.45 ^c^	0.29 ± 0.03 ^a^	Citrus, fresh floral, oily, sweet
***Aldehydes***								
2-methylpropanal	2.512	23.31 ± 0.03 ^c^	25.03 ± 0.13 ^d^	20.10 ± 0.23 ^a^	21.43 ± 0.21 ^ab^	24.3 ± 0.33 ^d^	22.14 ± 0.27 ^bc^	Wine, solvent, malty, fruity
3-methylbutanal	3.337	17.12 ± 0.33 ^d^	13.63 ± 0.36 ^b^	19.20 ± 0.21 ^e^	14.90 ± 0.15 ^bc^	15.08 ± 0.17 ^c^	4.52 ± 0.11 ^a^	Dried fruits, nutty, chocolate, cocoa, fatty
2-methylbutanal	3.469	15.80 ± 0.34 ^e^	9.22 ± 0.55 ^b^	16.44 ± 0.23 ^f^	13.8 ± 0.13 ^d^	11.56 ± 0.23 ^c^	5.18 ± 0.22 ^a^	Malty, cocoa, chocolate, coffee, caramellic, malty, nutty, rummy
hexanal	6.468	17.34 ± 0.13 ^a^	20.29 ± 0.13 ^b^	17.00 ± 0.42 ^a^	16.85 ± 0.07 ^a^	16.18 ± 0.12 ^a^	32.03 ± 0.15 ^c^	Intense green, aldehydic, fruity, oily
benzaldehyde	13.77	1.80 ± 0.04 ^cd^	1.27 ± 0.02 ^b^	2.14 ± 0.04 ^d^	1.33 ± 0.21 ^b^	1.47 ± 0.15 ^bc^	0.52 ± 0.02 ^a^	Almond, burnt sugar
Total		75.37 ± 1.04 ^d^	69.44 ± 1.28 ^c^	74.88 ± 1.03 ^d^	68.31 ± 0.79 ^b^	68.59 ± 0.88 ^bc^	64.39 ± 0.77 ^a^	
***Ketones***								
2-Heptanone	10.469	5.17 ± 0.23 ^ab^	7.91 ± 0.14 ^de^	4.28 ± 0.33 ^a^	6.31 ± 0.11 ^bc^	6.88 ± 0.21 ^cd^	8.60 ± 0.21 ^e^	Cheesy, fruity, spicy, creamy
Acetophenone	18.403	2.47 ± 0.03 ^a^	4.13 ± 0.03 ^c^	2.26 ± 0.05 ^a^	3.38 ± 0.03 ^b^	4.36 ± 0.03 ^c^	6.59 ± 0.13 ^d^	Floral, must, spicy, almond, nutty
Total		7.64 ± 0.26 ^b^	12.04 ± 0.17 ^d^	6.54 ± 0.38 ^a^	9.69 ± 0.14 ^c^	11.24 ± 0.24 ^d^	15.19 ± 0.34 ^d^	
***Esters***								
Butyl butyrate	15.459	3.32 ± 0.03 ^c^	2.09 ± 0.02 ^a^	4.07 ± 0.04 ^d^	2.80 ± 0.04 ^b^	2.10 ± 0.04 ^a^	1.86 ± 0.22 ^a^	Tropical fruits, woody, sweet, floral, green
Hexyl acetate	16.183	3.20 ± 0.17 ^b^	1.99 ± 0.21 ^a^	2.61 ± 0.04 ^ab^	2.00 ± 0.05 ^a^	2.05 ± 0.02 ^a^	10.81 ± 0.12 ^c^	Woody, green, pine
Total		6.52 ± 0.20 ^b^	4.08 ± 0.23 ^a^	6.69 ± 0.08 ^b^	4.80 ± 0.09 ^a^	4.15 ± 0.06 ^a^	12.67 ± 0.34 ^c^	
***Terpene and Terpenoids***								
Limonene	16.774	2.01 ± 0.03 ^c^	1.39 ± 0.01 ^b^	2.06 ± 0.03 ^c^	1.83 ± 0.22 ^bc^	1.47 ± 0.11 ^a^	0.18 ± 0.01 ^a^	Citrus, fresh, sweet
***Others***								
Dimethyl disulfide	4.393	2.21 ± 0.02 ^b^	3.75 ± 0.11 ^cd^	0.64 ± 0.02 ^a^	2.78 ± 0.03 ^bc^	3.19 ± 0.03 ^c^	3.20 ± 0.11 ^c^	Vegetal, sulfurous, cabbage, malt, cream
2-pentylfuran	15.085	1.51 ± 0.02 ^ab^	4.36 ± 0.02 ^c^	3.24 ± 0.31 ^bc^	3.76 ± 0.11 ^c^	4.68 ± 0.22 ^d^	1.29 ± 0.01 ^a^	Green, earthy, beans, musty, cooked, caramellike
Benzoic acid	22.276	3.43 ± 0.22 ^bc^	2.99 ± 0.02 ^abc^	3.71 ± 0.03 ^c^	4.73 ± 0.04 ^d^	2.28 ± 0.03 ^a^	2.79 ± 0.03 ^ab^	Balsamic
Total		7.15 ± 0.26 ^b^	11.1 ± 0.15 ^d^	7.59 ± 0.36 ^b^	11.27 ± 0.18 ^d^	10.15 ± 0.28 ^c^	2.28 ± 0.15 ^a^	

Different small letter in a row indicates the significant difference between samples (*p* < 0.05); values are presented as means of three determinations ± SD; BSG (brewers spent grains); BC—cookies sample manufactured with 100% WF (wheat flour); B1—cookies sample manufactured with 20% BSG1; B2—cookies sample manufactured with 20% BSG2; B3—cookies sample manufactured with 20% BSG3; B4—cookies sample manufactured with 20% BSG4; B5—cookies sample manufactured with 20% BSG5.

**Table 8:** Color and physical characteristic of cookies.

	Color Parameters			Physical Parameters			
Samples	L*	a*	b*	Weight (g)	Thickness (cm)	Diameter (cm)	Spread Factor (cm)
BC	76.21 ± 0.86 ^e^	6.04 ± 0.45 ^a^	39.03 ± 0.64 ^e^	13.01 ± 0.02 ^a^	1.01 ± 0.02 ^d^	5.20 ± 0.33 ^a^	5.20 ± 0.35 ^a^
B1	57.44 ± 0.66 ^b^	8.08 ± 0.41 ^b^	25.31 ± 0.43 ^b^	14.21 ± 0.04 ^b^	0.88 ± 0.03 ^ab^	5.30 ± 0.21 ^a^	6.02 ± 0.24 ^cd^
B2	62.39 ± 0.48 ^cd^	10.21 ± 0.61 ^d^	32.42 ± 0.54 ^d^	14.03 ± 0.02 ^b^	0.95 ± 0.02 c	5.25 ± 0.11 ^a^	5.52 ± 0.13 ^b^
B3	52.29 ± 0.81 ^a^	7.47 ± 0.32 ^b^	22.68 ± 0.43 ^a^	14.05 ± 0.05 ^b^	0.85 ± 0.04 ^a^	5.35 ± 0.31 ^a^	6.29 ± 0.35 ^d^
B4	60.95 ± 0.86 ^c^	9.32 ± 0.54 ^c^	28.73 ± 0.68 ^c^	14.11 ± 0.04 ^b^	0.90 ± 0.02 ^b^	5.40 ± 0.09 ^a^	6.00 ± 0.11 ^cd^
B5	63.45 ± 0.77 ^d^	10.34 ± 0.32 ^d^	32.61 ± 0.64 ^d^	14.13 ± 0.06 ^b^	0.92 ± 0.04 ^bc^	5.35 ± 0.04 ^a^	5.94 ± 0.08 ^c^

Different letters in a column indicates significant differences between samples; lightness (L*), redness (a*), yellowness (b*); BSG (brewers spent grains); BC—cookies sample manufactured with 100% WF (wheat flour); B1—cookies sample manufactured with 20% BSG1; B2—cookies sample manufactured with 20% BSG2; B3—cookies sample manufactured with 20% BSG3; B4—cookies sample manufactured with 20% BSG4; B5—cookies sample manufactured with 20% BSG5.

**Table 9:** Sensory evaluation of cookies.

Samples	Appearance	Hardness	Crispiness	Chewiness	Taste and Aroma	Overall Appreciation
BC	7.85 ± 0.51 ^a^	7.73 ± 0.17 ^bc^	7.39 ±0.22 ^a^	7.72 ± 0.31 ^a^	7.91 ± 0.02 ^b^	7.78 ± 0.02 ^a^
B1	8.01 ± 0.23 ^a^	7.90 ± 0.34 ^c^	8.15 ± 0.31 ^b^	7.90 ± 0.11 ^ab^	7.50 ± 0.03 ^a^	8.15 ± 0.03
B2	7.82 ± 0.55 ^a^	7.56 ± 0.22 ^a^	8.01 ± 0.41 ^b^	7.73 ± 0.42 ^a^	7.90 ± 0.02 ^b^	7.75 ± 0.12 ^a^
B3	8.90 ± 0.45 ^c^	8.10 ± 0.11 ^d^	8.45 ± 0.34 ^c^	7.96 ± 0.21 ^ab^	9.03 ± 0.02 ^d^	8.97 ± 0.04 ^b^
B4	8.51 ± 0.33 ^b^	7.99 ± 0.56 ^c^	8.35 ± 0.11 ^c^	7.82 ± 0.55 ^a^	8.58 ± 0.04 ^c^	8.17 ± 0.03 ^a^
B5	7.93 ± 0.22 ^a^	8.05 ± 0.12 ^c^	7.95 ± 0.33 ^b^	8.01 ± 0.63 ^b^	8.10 ± 0.05 ^b^	7.94 ±0.02 ^a^

Different small letter in a column indicates the significant difference between samples (*p* < 0.05); values are presented as means of three determinations ± SD; BSG (brewers spent grains); BC—cookies sample manufactured with 100% WF (wheat flour); B1—cookies sample manufactured with 20% BSG1; B2—cookies sample manufactured with 20% BSG2; B3—cookies sample manufactured with 20% BSG3; B4—cookies sample manufactured with 20% BSG4; B5—cookies sample manufactured with 20% BSG5.

**Table 10:** BSG (brewers spent grains) samples codification, type of malt used and kilning temperature.

BSG Samples Codes	Malt Type	Percent (%)	Approximate Kilning Temperature (°C)
BSG 1	Pilsner	78%	80–85
	Caramunich	20%	220
	Carafa	2%	250
BSG 2	Pale Ale 2RS	100%	80–85
BSG 3	Munich	70%	100–105
	Cara Gold	18%	220
	Melano	8%	130
	Chateau Black	4%	235
BSG 4	Pale Ale	56%	80–85
	Melano	26%	130
	Munich	10%	100–105
	Cara Gold	8%	220
BSG 5	Pale Ale	88%	90–95
	Cara Amber	6%	220
	Cara Blond	6%	220

**Table 11:** Cookie’s ingredients and technological parameters.

Ingredients (%)	Biscuits Samples					
	BC	B20%	B20%	B20%	B20%	B20%
Wheat flour (WF)	47.28	37.83	37.83	37.83	37.83	37.83
Brewers spent grain (BSG)	-	9.46	9.46	9.46	9.46	9.46
Butter	28.37	28.37	28.37	28.37	28.37	28.37
Eggs	14.18	14.18	14.18	14.18	14.18	14.18
Sugar powdered	9.46	9.46	9.46	9.46	9.46	9.46
Sodium bicarbonate	0.47	0.47	0.47	0.47	0.47	0.47
Salt	0.24	0.24	0.24	0.24	0.24	0.24
**Technological parameters**						
Mixing time (minutes)	8	8	8	8	8	8
Dough temperature (°C)	21	21.5	21.3	22.0	21.5	21.7
Resting time (minutes)	50	50	50	50	50	50
Temperature (°C)	4–5	4–5	4–5	4–5	4–5	4–5
Baking time (minutes)	12	12	12	12	12	12
Temperature (°C)	200	200	200	200	200	200

BC—cookies sample manufactured with 100% WF (wheat flour); BSG (brewers spent grains); B1—cookies sample manufactured with 20% BSG1; B2—cookies sample manufactured with 20% BSG2; B3—cookies sample manufactured with 20% BSG3; B4—cookies sample manufactured with 20% BSG4; B5—cookies sample manufactured with 20% BSG5.

## Data Availability

The data presented in this study are available on request from the corresponding author.
